# Dual-modal LAMP platform integrating biosensor and real-time fluorescence for rapid differentiation of *Mycobacterium tuberculosis* complex from nontuberculous mycobacteria

**DOI:** 10.1128/jcm.00662-26

**Published:** 2026-09-21

**Authors:** Junfei Huang, Xiaoyu Wei, Yonghu Wan, Chao Yang, Fengming Chen, Lu Zou, Yijiang Chen, Jing Chen, Xiu He, Shijun Li, Mingdan You, Guanghong Yang

**Affiliations:** 1 School of Public Health, the Key Laboratory of Environmental Pollution Monitoring and Disease Control, Ministry of Education, Guizhou Medical University, Guiyang, Guizhou, China; 2 Guizhou Provincial Center for Disease Control and Prevention, Guiyang, Guizhou, China; 3 State Key Laboratory of Functions and Applications of Medicinal Plants, Guizhou Provincial Engineering Technology Research Center for Chemical Drug R&D, Guizhou Medical University 74628, Guiyang, Guizhou, China; 4 Department of Nosocomial Infection Control, Guizhou Provincial People’s Hospital 56663 , Guiyang, Guizhou, China; The University of North Carolina at Chapel Hill School of Medicine, Chapel Hill, North Carolina, USA

**Keywords:** *Mycobacterium tuberculosis *complex, nontuberculous mycobacteria, loop-mediated isothermal amplification, nanoparticle-based lateral flow biosensor, real-time fluorescence

## Abstract

*Mycobacterium tuberculosis* complex (MTBC), the primary causative agent of pulmonary tuberculosis (PTB), shares pathological features with nontuberculous mycobacteria (NTM). Some NTM species act as opportunistic pathogens that can cause NTM lung disease, often leading to diagnostic confusion with PTB. Given the persistent global burden of tuberculosis and the rising incidence of NTM infections worldwide, there is an urgent need for rapid, highly sensitive diagnostic techniques capable of distinguishing MTBC and NTM at initial clinical presentation. In this study, we developed a novel diagnostic platform that integrates loop-mediated isothermal amplification (LAMP) with nanoparticle-based lateral flow biosensors (LFB), and real-time (RT) fluorescence analysis for simultaneous detection of MTBC and NTM. Target-specific primers were designed for *IS6110* (MTBC-specific) and 16S rDNA (pan-mycobacterial), and the corresponding probes were multi-labeled: FAM-biotin/digoxigenin-biotin for LFB capture, and FAM-BHQ1/Cy5-BHQ2 for real-time fluorescence monitoring. After systematic optimization, the LFB–RT–LAMP assay performed best at 67°C for 50 min. Analytical evaluation using genomic DNA from MTB reference strain H37Rv, 22 mycobacterial strains, 13 clinically relevant non-mycobacterial pathogens, and 80 respiratory specimens showed the following: (i) a limit of detection (LoD) of 576 fg/reaction for purified MTB DNA; (ii) 100% detection rate for the tested mycobacteria; (iii) 100% specificity against non-target pathogens; (iv) 100% clinical concordance with composite diagnostic PCR-based methods; and (v) a turnaround time of <60 min for both LFB readout and real-time fluorescence monitoring. Our dual-modal LFB–RT–LAMP platform provides a diagnostic workflow for simultaneous MTBC detection (via *IS6110*) and NTM screening (via 16S rDNA) in a single assay. The method achieves a turnaround time of under 60 min, a sensitivity of 576 fg/reaction, and 100% specificity against non-mycobacterial pathogens, demonstrating potential for point-of-care use in clinical and field settings.

**IMPORTANCE:**

Tuberculosis (TB) and nontuberculous mycobacteria (NTM) lung disease present with similar symptoms but require completely different treatment regimens. Rapid and accurate differentiation between these pathogens at initial presentation remains a critical unmet need, as current molecular tests primarily focus on TB and are not designed for NTM screening. In this study, we developed a simple, rapid, and equipment‑free diagnostic platform that distinguishes TB from NTM within 60 min using a lateral flow biosensor, alongside a real‑time fluorescence option for laboratory use. The assay simultaneously targets *IS6110* (*Mycobacterium tuberculosis* complex [MTBC]‑specific) and 16S rDNA (pan‑mycobacterial), enabling species‑level identification of MTBC and genus‑level screening of NTM in a single reaction, achieving 100% accuracy on clinical samples with a limit of detection of 576 fg (~120 genome copies) per reaction. This dual‑modal approach offers practical solutions for both well‑equipped hospitals and remote clinics, addressing a critical gap in TB and NTM diagnostics.

## INTRODUCTION

Tuberculosis (TB), caused by *Mycobacterium tuberculosis* complex (MTBC), persists as the leading cause of infectious disease mortality despite being preventable and curable (1, 2). While pulmonary tuberculosis (PTB) is the most common clinical manifestation, the epidemic’s scale remains staggering—the WHO reported 10.7 million incident cases and 1.23 million deaths in 2024 alone (2). Excluding the MTB complex and *Mycobacterium leprae*, over 190 species of environmental mycobacteria are classified as nontuberculous mycobacteria (NTM) (3). While some are saprophytic, clinically relevant pathogens—including *M. abscessus*, the *M. avium* complex, and *M. kansasii—*can cause severe pulmonary and extrapulmonary disease in immunocompromised hosts (3, 4). Clinical presentations range from cystic fibrosis exacerbations and hypersensitivity syndromes to skin/soft tissue infections (3–5). Among NTM-associated pathologies, pulmonary disease (NTM-PD) is the most common clinical presentation (4, 6). Its global incidence and prevalence are rising (7). Untreated NTM-PD carries a significant mortality risk—exemplified by the approximately 33% fatality rate for *M. avium* complex (8). Because treatment regimens for NTM differ substantially from those for PTB, these trends underscore the urgent need for rapid diagnostics capable of at least genus-level NTM identification to guide further species-specific testing.

PTB and NTM-PD present with overlapping symptoms—cough, fever, sputum production, hemoptysis, and cavitary lesions—yet their epidemiological profiles differ markedly (5, 9, 10). However, conventional diagnostics relying on culture and acid-fast staining fail to discriminate these phylogenetically related pathogens due to morphological and biochemical similarities (6, 7). Nucleic acid amplification (NAA) assays have improved MTBC detection but rarely identify NTM concurrently, especially in early-stage disease (7). This is clinically problematic: culture-positive or acid-fast stains from symptomatic patients more often indicate MTB infection than NTM colonization (10, 11). With TB causing more than 10 million cases annually versus NTM-PD’s rising opportunistic burden, novel NAA platforms capable of simultaneous MTBC/NTM screening are urgently needed.

Loop-mediated isothermal amplification (LAMP), first described by Notomi et al. (12), is a nucleic acid amplification technique characterized by operational simplicity, rapid turnaround, and high specificity (12, 13). This method amplifies target sequences at constant temperatures (60°C–70°C), generating >10⁹ DNA copies within 60 min through autocycling strand displacement (13, 14). Key to this process, Bst DNA polymerase is an enzyme with intrinsic strand-displacement activity that eliminates the thermal denaturation requirement of conventional PCR (12–14). LAMP amplicon detection employs multiple platforms, each with distinct advantages: colorimetric indicators (e.g., hydroxynaphthol blue), nanoparticle-based lateral flow biosensors (LFB), real-time turbidimetry, and fluorometric monitoring (15, 16). These integrated systems enable visual or instrumental readout, facilitating point-of-care (POC) deployment (13, 15, 17). Thus, LAMP has become a valuable tool for molecular diagnostics in clinical microbiology, particularly in resource-limited settings (13, 16–19).

To resolve the diagnostic challenge of concurrent MTBC and NTM detection, we developed a dual-target LAMP system integrating *IS6110* (MTBC-specific) and 16S rDNA (pan-mycobacterial) primers. LAMP platforms using colorimetric or turbidimetric readouts lack multiplex discrimination (15, 20). Although LAMP combined with LFB or fluorescence systems supports multi-analyte detection individually (16, 21), no current system integrates both modalities, limiting their applicability across diverse settings. Our LFB–RT–LAMP platform bridges this gap with a bi-functional probe system: (i) biotinylated capture probes with FAM/digoxigenin reporters for LFB detection and (ii) fluorescence probes (FAM-BHQ1/Cy5-BHQ2) for real-time monitoring. Performance was validated via serial MTB DNA dilution (limit of detection [LoD] testing), a panel of relevant pathogens (accuracy testing), and respiratory specimens (clinical accuracy). This system provides a field-deployable, instrument-flexible MTBC/NTM detection solution with significant potential for point-of-care support in cases with overlapping symptoms.

## MATERIALS AND METHODS

### Design and synthesis of dual-target LAMP primers for dual-detection system

We targeted the MTBC-specific *IS6110* gene (GenBank: X98149.1) and pan-mycobacterial 16S rDNA (GenBank: MH794239.1) after BLAST-based alignment and specificity screening. Three sets of primers (F3/B3, FIP/BIP, and LF/LB) were designed via PrimerExplorer V5 (Eiken Chemical) for each gene. HPLC-purified primers were synthesized by Sangon Biotech (Shanghai, China). The set with the highest amplification efficiency was identified via real-time turbidimetry (LA-500, Eiken Chemical) and selected for dual-labeling. Real-time turbidimetry measures the increase in turbidity caused by the precipitation of magnesium pyrophosphate, a byproduct of DNA polymerization, which correlates with amplicon accumulation. During amplification, the instrument automatically collects turbidity signals every 6 s at the set temperature and displays a real-time kinetic curve.

Our dual-detection system comprised the following. (i) For LFB detection, the unmodified loop primers were replaced with IS6110-LB# (FAM-labeled) and 16S-LB# (Dig-labeled) primers. During LAMP amplification in the presence of biotin-dCTP, these primers generated FAM-biotin and Dig-biotin amplicons, which are specifically captured and visualized on the LFB. (ii) For real-time fluorescence detection, the IS6110-LB* (FAM-BHQ1) and 16S-LF* (Cy5-BHQ2) primers function as fluorogenic probes. During primer extension and strand-displacement DNA synthesis, the released fluorophore from its quencher yields a measurable fluorescence signal. All primer sequences are detailed in Table 1, with their binding positions illustrated in Fig. S1.

**TABLE 1:** Primer details for the *IS6110* and 16S rDNA target genes^*a*^

Gene	Primers	Sequences and modifications (5′→3′)	Length
*IS6110*	IS6110-F3	AGTACAGCCACACCTTTGTG	20 nt
	IS6110-B3	ACCCGCACCACCTGCT	16 nt
	IS6110-FIP	ATGATGGCTGTGGCGTGCCC-CGAAGTGGGTCGCCAACT	38 mer
	IS6110-BIP	GCCCGTGAACAGTTCGGCCT-AGGTCACGCTCCAGACG	37 mer
	IS6110-LF	CCTTGACGGTCTTCTCACCG	20 nt
	IS6110-LB	ATCGAGGACTGCCGCAACATG	21 nt
	IS6110-LB#	AT(FAM)CGAGGACTGCCGCAACATG	21 nt
	IS6110-LB*	AT(FAM)CGAGGACTGCCGCAACAT(BHQ1)G	21 nt
*16S DNA*	16S-F3	TTAACTGTGAGCGTGCGG	18 nt
	16S-B3	GGAAGGAAACCCACACCTAG	20 nt
	16S-FIP	GCCACCGGTGTTCCTCCTGATA-GACTAGAGTACTGCAGGGGA	42 mer
	16S-BIP	GCGGGTCTCTGGGCAGTAAC-GCGTGGACTACCAGGGTAT	39 mer
	16S-LF	GCTACACCAGGAATTCCAGTC	21 nt
	16S-LB	TGACGCTGAGGAGCGAAAG	19 nt
	16S-LB#	Digoxin-TGACGCTGAGGAGCGAAAG	19 nt
	16S-LF*	GCT(Cy5)ACACCAGGAATTCCAGT(BHQ2)C	21 nt

^*a*^ F3 and B3 denote the forward and reverse outer primers, respectively. FIP (forward inner primer) consists of the F1c sequence (in reverse complement) linked to F2. BIP (backward inner primer) consists of B1c linked to the reverse complement of B2. LF and LB are loop primers. The primers were modified as follows: IS6110-LF# (5′-FAM), IS6110-LF* (5′-FAM, 3′-BHQ1), 16S-LB# (5′-Digoxin), and 16S-LF* (5′-Cy5, 3′-BHQ2). FAM, carboxyfluorescein; Cy5, cyanine 5; BHQ, black hole quencher; mer, monomeric unit; nt, nucleotide.

### Construction of the nanoparticle-based LFB

**Fig 1:**
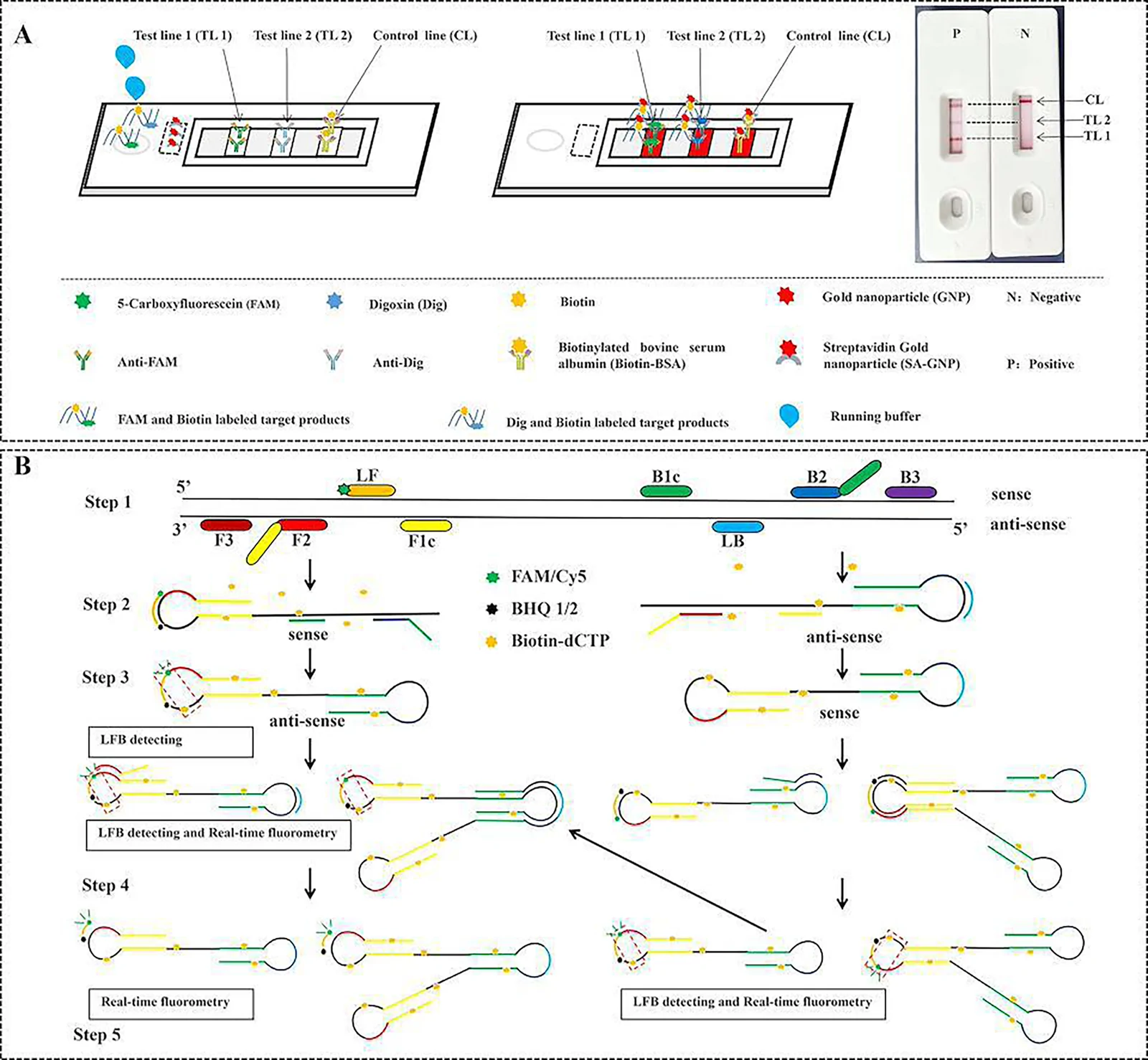
(**A**) Schematic of the LFB construct for simultaneous detection of IS6110 (MTB) and 16S rDNA (pan-mycobacterium). For detailed assembly, reaction conditions, and interpretation of results, see Materials and Methods (Construction of the nanoparticle-based LFB). (**B**) LFB–RT–LAMP detection mechanism. Step 1: Bst DNA polymerase extends primers on the target template, incorporating biotin-dCTPs. Steps 2 and 3: The FAM-BHQ1/Cy5-BHQ2–labeled loop primer (LF/LB) extends, creating a dual-label amplicon (FAM-biotin/Dig-biotin) detectable by LFB. Steps 4 and 5: Strand displacement releases the FAM/Cy5 fluorophore from BHQ1/BHQ2 quenching, enabling real-time fluorescence monitoring.

A nanoparticle-based lateral flow biosensor (LFB) was constructed by laminating four functional components—sample pad, conjugate pad, nitrocellulose membrane, and absorbent pad—onto a plastic backing card (22, 23). The membrane was pre-coated with anti-FAM antibody (test line 1, TL1), anti-digoxigenin antibody (TL2), and biotinylated bovine serum albumin (control line, CL) through precision dispensing. For detection, 1.0–1.5 µL of dual-target LAMP amplicons was applied to the sample pad followed by 150–200 µL running buffer. Amplified products complexed with streptavidin-conjugated gold nanoparticles migrated laterally, forming visible complexes at corresponding capture lines (Fig. 1A). Customized LFBs were manufactured by Tianjin HuiDeXin Biotech (China) per design specifications. MTB-positive: red bands at TL1 (*IS6110*), TL2 (16S rDNA), and CL; NTM positive: bands at TL2 and CL; Negative: only CL band.

### Bacterial strains and clinical samples

We tested 22 mycobacterial strains (covering *Mycobacterium tuberculosis*, *Mycobacterium leprae*, and prevalent NTM species) and 13 non-mycobacterial pathogens (*Klebsiella pneumoniae*, *Streptococcus suis*, *Bacillus anthracis*, *Staphylococcus aureus*, etc.) from the Guizhou Provincial CDC. For clinical validation, we included 45 sputum proficiency panels (Chinese CDC, 2022–2024) and 35 clinical sputum specimens (Guizhou Province, February–October 2022) to mirror both diagnostic performance and routine diagnostic workflows. All procedures using pathogenic strains and clinical specimens were performed in a BSL-2 laboratory following standard biosafety protocols.

### DNA extraction of strains and clinical samples

Cultured strains were harvested with sterile disposable loops and transferred to 1.5 mL tubes containing 0.5 mL 1× TE buffer (pH 8.0). Bacteria were inactivated at 80°C for 30 min in a heating block. After centrifugation at 12,000 rpm for 5 min (centrifugal radius, 7.5 cm), supernatants were discarded. Sputum specimens were digested with 4% NaOH (room temperature, 15 min) in 5 mL tubes, centrifuged at 6,000 rpm for 10 min, and supernatants were removed. Pellets were resuspended in 0.5 mL TE buffer and processed as above.

We extracted genomic DNA using a commercial kit (Tianlong Technology, China) following the optimized protocol: (i) enzymatic digestion with 180 μL lysozyme and 20 μL proteinase K at 50°C for 30 min with agitation; (ii) addition of 200 μL bacterial lysis buffer; and (iii) automated purification using a magnetic bead-based nucleic acid extraction system. Extracted DNA was stored at −20°C for subsequent analysis.

### LFB–RT–LAMP reaction

The LAMP primers targeting *IS6110* and 16S rDNA were validated through standardized LAMP reactions. Each 25 μL reaction mixture contained the following: 2.5 μL isothermal amplification buffer (10×), 1.3 μL MgSO₄ (7.2 mM final), 1.4 μL dNTPs (1.4 mM each), 1.0 μL biotin-14-dCTP (50 nmol), 1.1 μL primer mix (1.6 μM FIP/BIP, 0.4 μM LF/LB, and 0.2 μM F3/B3), 1 μL Bst DNA polymerase (8 U/μL), template DNA (1.0 μL for pure cultures; 2.5 μL for clinical samples), and DNase-free water to volume. All reagents were from Beyotime Biotechnology Co., Ltd. (Shanghai, China), except biotin-14-dCTP (Tianjin HuiDeXin, China).

We performed reactions at 65°C for 60 min and inactivated enzymes at 85°C for 5 min. Amplification products were verified by four methods: real-time turbidimetry (LA-500, Eiken Chemical Co., Ltd., Japan), agarose gel electrophoresis (Bio-Rad, USA), LFB detection (Tianjin HuiDeXin Biotech, China), and fluorometric monitoring (Tianlong Technology, China). Negative control used no-template (DNase-free water) reactions. For LFB–RT–LAMP, general primers were replaced with dual-functional labeled primers to generate the LAMP-labeled amplicons (biotinylated amplicons for LFB readout; fluorophore-quencher pairs for real-time detection), with primer concentrations adjusted proportionally.

### Optimization of the temperature and time for the LAMP reaction

Bst DNA polymerase usually operates at 65°C (12, 16, 20), but the differential GC content of our target genes—IS6110 (65.4%; X98149.1) and 16S rDNA (57.8%; MH794239.1)—may require temperature adjustment for efficient amplification. We therefore optimized the dual-target LAMP assay across 62°C–69°C (1°C increments) using *M. tuberculosis* H37Rv (ATCC 27294) genomic DNA (14.4 pg/μL) as a template. Amplification was monitored in real time by turbidimetry, with positivity defined as turbidity >0.1 absorbance units within 60 min (16, 22). Concurrently, reaction time was determined by evaluating the accumulated turbidimetry curve of generated amplicons.

### Verification of the limit of detection and accuracy of the LFB–RT–LAMP assay

We assessed analytical sensitivity using fivefold serial dilutions of *M. tuberculosis* H37Rv genomic DNA (72 pg/μL to 4.6 fg/μL). The limit of detection (LoD) was determined from six replicates at each dilution. Accuracy was evaluated against 35 bacterial strains under optimized conditions. Products were verified by LFB detection and real-time fluorescence. No-template controls (NTCs) with DNase-free water were included in all runs.

### Accuracy of the LFB–RT–LAMP assay for clinical samples

We validated the LFB–RT–LAMP assay clinically using 2.5 μL DNA extracts from prospectively collected specimens under optimized conditions. All samples were tested in parallel against the GeneXpert MTB/RIF system (Cepheid, USA), which served as the reference standard (supplemental methods). NTM-suspected samples were identified by real-time PCR melting curve analysis (Zeesan Biotech, China) (supplemental methods). *M. tuberculosis* H37Rv DNA and DNase-free water served as positive and negative controls, respectively.

Two sample groups were used: 45 sputum proficiency testing panels (artificial sputum with inactivated bacteria) from the Chinese CDC (2022–2024), consisting of MTB-positive, NTM-positive, and NTM-negative samples, designed primarily to assess the diagnostic performance of LFB–RT–LAMP; 35 sputum samples from Guizhou Province (February–October 2022) were used to evaluate clinical feasibility. Double-blind testing was performed on all specimens.

### Statistical analysis

Statistical analyses were performed using GraphPad Prism 10. LoD determination, the lowest concentration with ≥95% positive detection (≥19/20 replicates), was defined as the LoD. Linearity between Ct values and log₁₀-transformed DNA concentrations was assessed by linear regression. For clinical sensitivity and specificity, they were calculated with 95% confidence intervals (CIs) using the Wilson score method.

## RESULTS

### Schematic mechanism of the nanoparticle-based LFB capture and fluorescence monitoring

Integration of these complementary detection modalities—LFB visualization and real-time fluorometry—established the novel LFB–RT–LAMP platform, enabling simultaneous optical and instrumental signal acquisition (Fig. 1). To achieve dual-modal detection of the *IS6110* target, we designed the IS6110-LB* primer with a FAM-BHQ1 fluorophore-quencher pair. Amplification in the presence of biotin-dCTP yielded two parallel outputs: the incorporation of biotin produced FAM-biotin amplicons for LFB readout, and the strand displacement of the fluorophore-quencher pair provided a real-time fluorescence signal. For simultaneous detection of the 16S rDNA target, we implemented a complementary primer-labeling scheme. The 16S-LF* primer (Cy5-BHQ2) generated the real-time fluorescence signal, while the 16S-LB# primer (Dig) facilitated LFB detection via the formation of Dig-biotin amplicons during co-amplification with biotin-dCTP.

We hypothesize that the underlying mechanism is attributable to the strong strand-displacement activity of Bst DNA polymerase. During strand displacement between the loop primers and the amplicon region, the LF/LB primers labeled with the fluorophore-quencher pair are incorporated into or displaced from the DNA strand. This process interferes with the quenching efficiency of BHQ toward the fluorophore—specifically by attenuating the electron-withdrawing capacity of BHQ, thereby allowing the fluorescence to be emitted and subsequently detected by the instrument. As amplification products accumulate, the fluorescence signal progressively increases until it reaches a plateau phase.

### Confirmation and detection of LFB–RT–LAMP products

Validation of the LFB–RT–LAMP assay employed four detection methods: real-time turbidity, gel electrophoresis, LFB, and real-time fluorescence. Each reaction contained 14.4 pg of MTB genomic DNA, with DNase-free water as the NTC. The roles of the various labeled primers were confirmed according to the schemes in Table 2.

**TABLE 2:** Functions and performance of the labeled primers evaluated within the LAMP system^*a*^

No.	Method/labeled	Real-time turbidimeter	Agarose gel electrophoresis	LFB–RT–LAMP (LFB/real-time fluorescence)	
a	IS6110- none labeled	+	+	−	−
b	IS6110-LB# Biotin-dCTP	+	+	+	−
c	IS6110-LB*	+	+	−	+
d	IS6110-LB* Biotin-dCTP	+	+	+	+
e	16S- none labeled	+	+	−	−
f	16S-LB# Biotin-dCTP	+	+	+	−
g	16S-LF*	+	+	−	+
h	16S-LF* 16S-LB# Biotin-dCTP	+	+	+	+

^*a*^ The table outlines the primer sets and modifications used to validate the dual-detection LAMP system. Groups a and e: standard, unmodified LAMP primers. Groups b and f : the LB primer was replaced with a labeled version (LB#, specifics depend on the target gene), and biotin-dCTP was added to enable LFB detection. Group c: the LB primer was replaced with a fluorogenic probe (LB*, FAM-BHQ1) for real-time fluorescence detection of *IS611*0. Group g: the LF primer was replaced with a fluorogenic probe (LF*, Cy5-BHQ2) for real-time fluorescence detection of 16S rDNA. Group d: the LB primer was replaced with a fluorogenic probe (LB*), and biotin-dCTP was added for dual LFB and fluorescence detection of *IS6110*. Group h: combined modifications (LF*, LB#, and biotin-dCTP) for dual fluorescence and LFB detection of 16S rDNA. +, positive; −, negative.

**Fig 2:**
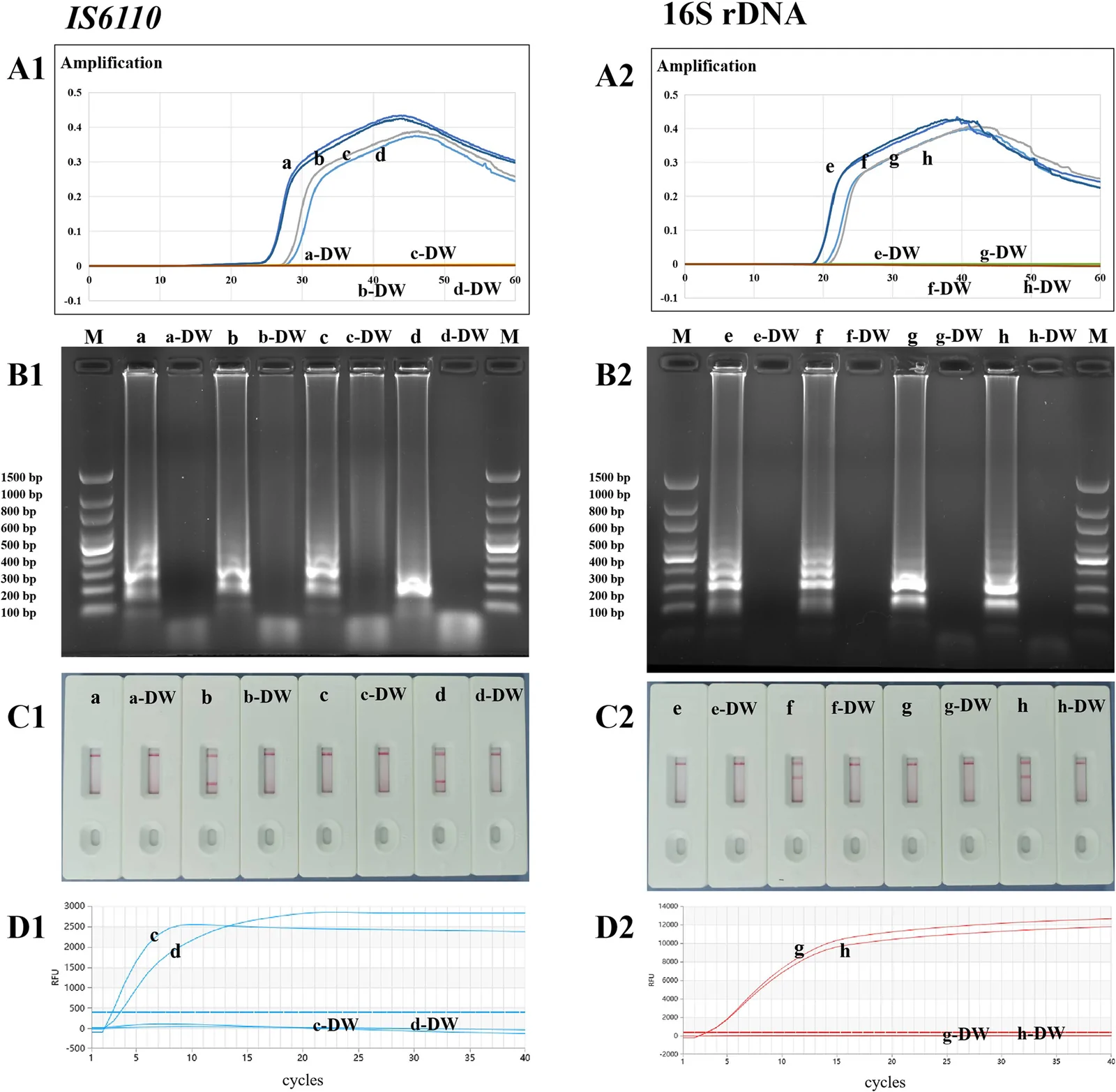
Validation of the LAMP assay with differentially labeled primers. Groups A1–D1 and A2–D2 display the detection results for the *IS6110* and 16S rDNA targets, respectively, using four independent methods. The specific detection methods are as follows: real-time turbidity monitoring (A1 and A2)**,** agarose gel electrophoresis (B1 and B2), LFB readout (C1 and C2), and real-time fluorescence monitoring (D1 and D2). Within each sub-panel, the results for the eight experimental groups (a to h) and their corresponding no-template controls (a-DW to h-DW) are presented (detailed in Table 2).

The performance of the LFB-RT-LAMP assay was first evaluated for each target gene (*IS6110* and 16S rDNA) individually. Successful amplification for all primer sets listed in Table 2 (groups a–h) was confirmed by both real-time turbidity monitoring (Fig. 2A1 and A2) and agarose gel electrophoresis (Fig. 2B1 and B2). For LFB detection, reactions required primers labeled with FAM or Digoxin (Dig) in combination with biotin-dCTP (Fig. 2C1-b and C2-f). In contrast, real-time fluorescence monitoring depended on primers incorporating fluorophore-quencher pairs (FAM-BHQ1 or Cy5-BHQ2) (Fig. 2D1-c and D2-g). Importantly, integrating both labeling strategies (FAM/Dig, biotin, and fluorophore-quencher pairs) into a unified one-pot reaction enabled the concurrent detection of amplicons by both LFB (Fig. 2C1-d and C2-h) and real-time fluorescence (Fig. 2D1-d and D2-h). An additional finding was that the IS6110-LB* primer (FAM-BHQ1) could functionally replace the IS6110-LB# primer (FAM) to generate “FAM-biotin” amplicons compatible with LFB detection, a mechanism accounted for in the assay schematic (Fig. 1B).

**Fig 3:**
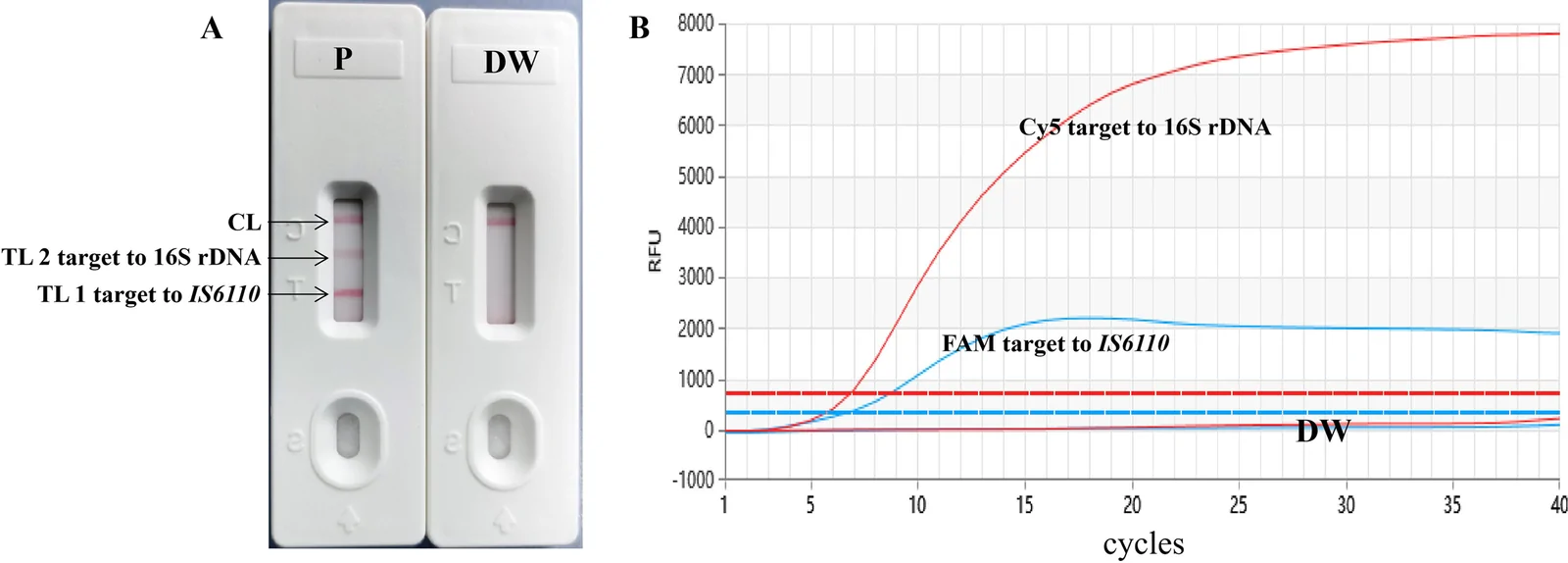
Validation of the dual-target LFB-RT-LAMP assay. (**A**) LFB readout. The test lines correspond to specific targets: TL1 for the *IS6110* gene, TL2 for the 16S rDNA gene, and CL for the control line. (**B**) Real-time fluorescence monitoring. Amplification curves are shown for each target: the *IS6110* gene (FAM channel, blue curve) and the 16S rDNA gene (Cy5 channel, red curve). In both panels, DW denotes the no-template control using DNase-free water.

Then, we established an optimized, dual-target LAMP assay in a one-pot reaction using a mixture of labeled primers (1.1 μL each of IS6110-specific and 16S rDNA-specific primers per reaction). Amplification products for both targets were concurrently detected by LFBs and real-time fluorescence, demonstrating the functionality of the integrated LFB–RT–LAMP platform (Fig. 3).

### Optimization of temperature and time for the dual LFB–RT–LAMP assay

**Fig 4:**
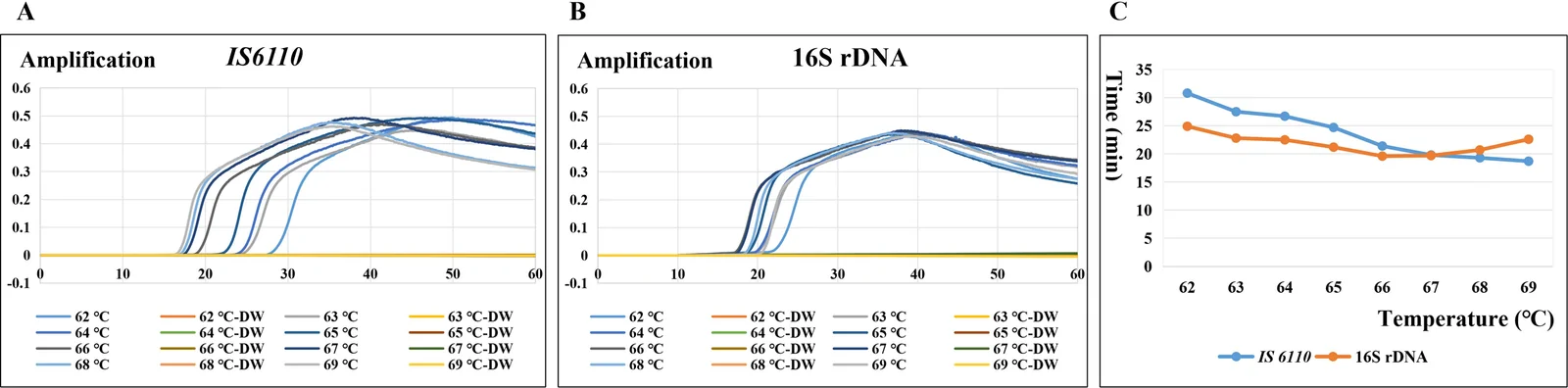
Optimization of temperature and time for the dual-target LFB–RT–LAMP assay. (**A and B**) Cumulative turbidity curves for the *IS6110* (**A**) and 16S rDNA (**B**) targets across a temperature gradient (62°C–69°C). (**C**) Threshold time plotted as a function of temperature for both targets, identifying the optimal reaction temperature.

Optimal reaction temperature and time for the dual-target LFB–RT–LAMP system required optimization. The amplification temperature was first screened across a range from 62°C to 69°C at 1°C intervals, using 14.4 pg of H37Rv genomic DNA per reaction and monitoring amplification via real-time turbidimetry. Both the *IS6110* and 16S rDNA targets exhibited efficient amplification (turbidity >0.1) across the entire temperature range tested, as indicated by robust turbidity curves (Fig. 4A and B). Further analysis of the threshold time versus temperature plot identified an intersection point at 67°C (Fig. 4C), where both targets demonstrated higher amplification efficiency, characterized by strong turbidity signals and an earlier time to reaction.

Based on these observations, the turbidity curves also indicated that the amplification reactions reached saturation before 45 min (Fig. 4A and B). To ensure robust and simultaneous amplification of both target genes while allowing for complete reaction, the optimal conditions for the LFB–RT–LAMP assay were determined to be 67°C for 50 min.

### Limit of detection of the LFB–RT–LAMP assay

**Fig 5:**
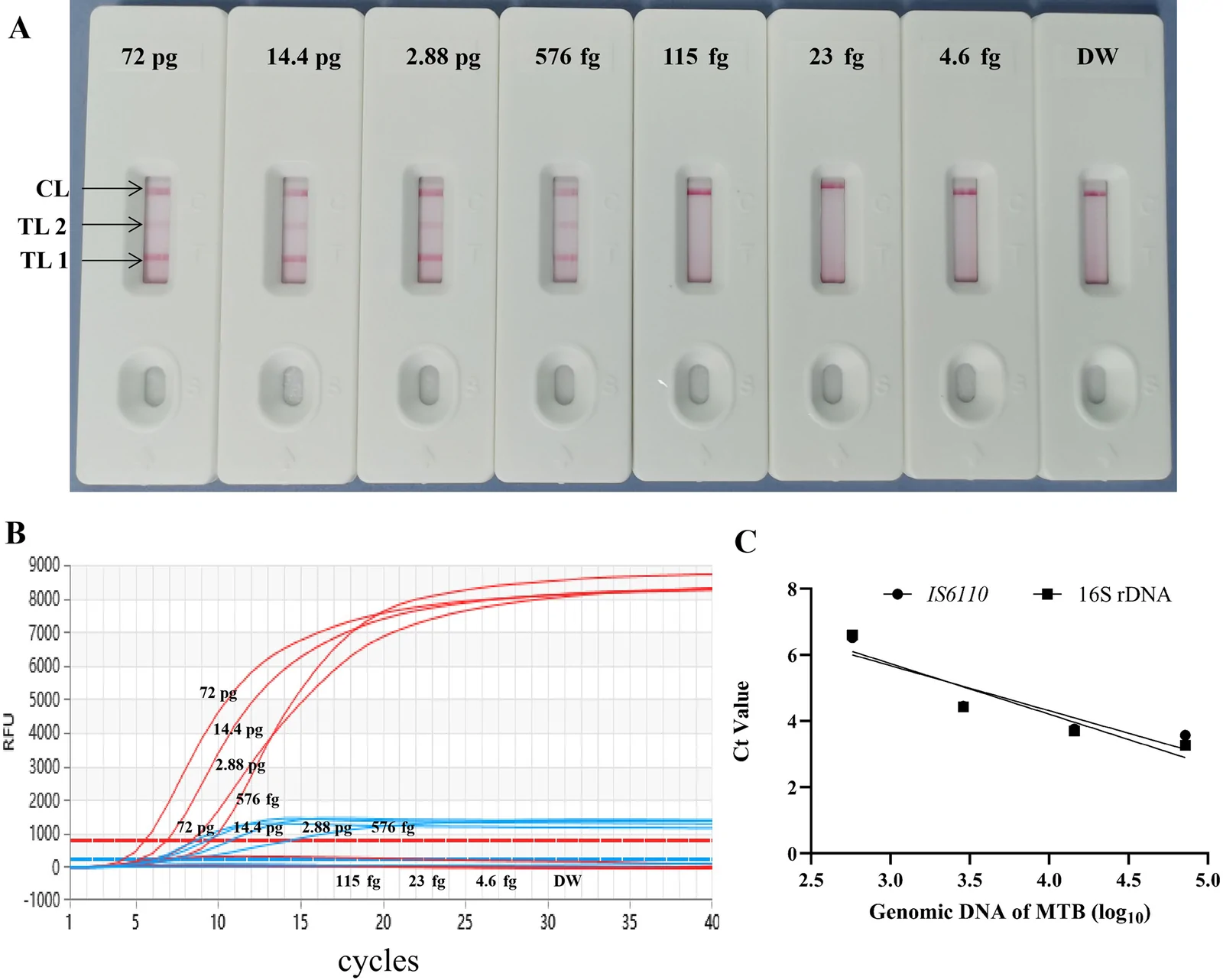
Analytical sensitivity of the dual-target LFB–RT–LAMP assay. (**A**) LFB detection. Results for a dilution series of genomic DNA are shown. Test line 1 (TL1) and test line 2 (TL2) indicate detection of the IS6110 and 16S rDNA targets, respectively. The CL verifies valid assay performance. (**B**) Real-time fluorescence monitoring. Amplification curves are shown for the IS6110 target (FAM channel, blue curve) and the 16S rDNA target (Cy5 channel, red curve). DW, DNase-free water (negative control). (**C**) Correlation between Ct values and DNA concentration. The Ct values of positive reactions are plotted against the log_10_-transformed DNA concentration (fg/reaction). The ordinate represents the threshold cycle number, and the abscissa represents the log_10_ of MTB DNA concentration.

The analytical sensitivity of the LFB–RT–LAMP assay was determined using fivefold serial dilutions of H37Rv genomic DNA, ranging from 72 pg/µL to 4.6 fg/µL. The LoD for each target (*IS6110* and 16S rDNA) was assessed individually. Both targets exhibited a consistent detection threshold of 115 fg DNA per reaction in preliminary triplicate experiments, as confirmed by LFB, real-time fluorescence, and agarose gel electrophoresis (Fig. S2). Subsequently, to evaluate the integrated dual-target assay, the LoD was determined to be 576 fg per reaction using both LFB and real-time fluorescence detection, with 20 replicates at each concentration (Fig. 5A and B). The lowest concentration tested with 100% detection (20/20) was 576 fg per reaction, whereas at 115 fg only 80% (16/20) positivity was observed. Given that one MTB genome is approximately 4.8 fg, the dual-target LoD of 576 fg corresponds to about 120 genome copies per reaction (4.8 copies/μL).

Moreover, the positive Ct values were plotted against the log_10_-transformed DNA concentrations over the detection range from 7.2 × 10⁵ fg to 5.76 × 10² fg (Fig. 5C), with the linear regression equations: *IS6110*, *Y* = −1.362*X* + 9.760 (*R*² =0.8319); 16S rDNA, *Y* = −1.530*X* + 10.33 (*R*² =0.8750).

### Accuracy of the dual LFB–RT–LAMP assay

The accuracy of the dual LFB–RT–LAMP assay was evaluated using genomic DNA from a panel of 35 pathogenic strains, comprising 22 mycobacterial and 13 non-mycobacterial species (Table 3). Moreover, prevalent NTM species vary by geographic region; we used six locally isolated mycobacterial strains from Guizhou Province (southwest China) for accuracy testing. As expected, *M. tuberculosis* generated positive signals for both the *IS6110* and 16S rDNA targets. The remaining 21 mycobacterial strains tested positive only for the 16S rDNA target (Fig. S3), while all 13 non-mycobacterial pathogens yielded negative results for both targets (Fig. S4). These data demonstrate a 100% detection rate for the tested mycobacteria and 100% specificity for the non-target pathogens. DNase-free water was included as a NTC in all accuracy analyses.

**TABLE 3:** Strain details and LFB-RT-LAMP detection results^*a*^

Bacteria	Source of strains	No. of strains	LFB		Real-time fluorescence	
			TL1	TL2	FAM	Cy5
Mycobacterium						
*M. tuberculosis*	H37Rv, ATCC27294	1	+	+	+	+
*M. kansasii*	ATCC12478	1	−	+	−	+
*M. intracellulare*	ATCC13950	1	−	+	−	+
*M. chelonae*	ATCC14472	1	−	+	−	+
*M. neoaurum*	ATCC25795	1	−	+	−	+
*M. marinum*	ATCC927	1	−	+	−	+
*M. gilvum*	ATCC43909	1	−	+	−	+
*M. microti*	ATCC19422	1	−	+	−	+
*M. vaccae*	ATCC15483	1	−	+	−	+
*M. phlei*	ATCC11758	1	−	+	−	+
*M. scrofulaceum*	ATCC19981	1	−	+	−	+
*M. gastri*	ATCC15754	1	−	+	−	+
*M. xenopi*	ATCC19970	1	−	+	−	+
*M. abscessus*	ATCC19977	1	−	+	−	+
*M. ulcerans*	ATCC19423	1	−	+	−	+
*M. malmoense*	ATCC29571	1	−	+	−	+
*M. leprae*	Isolate strains (GZCDC)	1	−	+	−	+
*M. avium*	Isolate strains (GZCDC)	1	−	+	−	+
*M. intracellulare*	Isolate strains (GZCDC)	1	−	+	−	+
*M. fortuitum*	Isolate strains (GZCDC)	1	−	+	−	+
*M. chelonae*	Isolate strains (GZCDC)	1	−	+	−	+
*M. smegmatis*	Isolate strains (GZCDC)	1	−	+	−	+
Non-mycobacterium						
*Brucella melitensis*	Isolate strains (GZCDC)	1	−	−	−	−
*Klebsiella pneumoniae*	Isolate strains (GZCDC)	1	−	−	−	−
*Haemophilus influenzae*	Isolate strains (GZCDC)	1	−	−	−	−
*Streptococcus pneumoniae*	Isolate strains (GZCDC)	1	−	−	−	−
*Staphylococcus aureus*	Isolate strains (GZCDC)	1	−	−	−	−
*Nontyphoidal Salmonella*	Isolate strains (GZCDC)	1	−	−	−	−
*Pseudomonas aeruginosa*	Isolate strains (GZCDC)	1	−	−	−	−
*Bacillus anthracis*	Isolate strains (GZCDC)	1	−	−	−	−
*Streptococcus suis*	Isolate strains (GZCDC)	1	−	−	−	−
*Neisseria gonorrhoeae*	Isolate strains (GZCDC)	1	−	−	−	−
*Shigella dysenteriae*	Isolate strains (GZCDC)	1	−	−	−	−
HIV-1	Isolate strains (GZCDC)	1	−	−	−	−
H1N1	Isolate strains (GZCDC)	1	−	−	−	−

^*a*^ ATCC, American Type Culture Collection; GZCDC, Guizhou Provincial Center for Disease Control and Prevention. Targets: *IS6110* (TL1/FAM), 16S rDNA (TL2/Cy5). +, positive; −, negative. MTB positive: both targets detected. NTM positive: only 16S rDNA detected.

To evaluate assay performance in co-infection scenarios, we tested mixtures of MTB H37Rv and *M. avium* (MA) DNA. Two MTB concentrations were tested—72 pg/reaction and 576 fg/reaction (the LoD of the dual-target assay), each mixed with MA DNA. As shown in Fig. 5, in all co-infection samples, both *IS6110* and 16S rDNA targets were detected, confirming MTBC positivity. The presence of MA did not interfere with MTB detection at either high or low MTB concentrations. The current format cannot distinguish MTB-alone from MTB-NTM co-infection because the pan-mycobacterial target is positive for both.

### Practical application of the dual LFB–RT–LAMP assay

Compared to the GeneXpert MTB/RIF system, the dual LFB–RT–LAMP assay demonstrated identical performance on proficiency testing panels, correctly identifying 22 MTBC-positive and 23 MTBC-negative samples. When applied to clinical samples, the assay achieved 100% accuracy, correctly classifying 31 MTBC-positive and 4 MTBC-negative cases (Table 4). Pooled analysis of proficiency panels (*n*_1_ = 45) and clinical samples (*n*_2_ = 35) yielded a sensitivity of 100% (95% CI: 93.3%–100%) and a specificity of 100% (95% CI: 87.2%–100%) for MTB identification.

**TABLE 4:** Detection results of two sample groups by GeneXpert MTB/RIF and LFB-RT-LAMP

Sample groups/method	GeneXpert MTB/RIF MTBC	Dual LFB–RT–LAMP	
		MTBC	NTM
Proficiency testing panels (*n*_1_ = 45)			
MTBC positive	22	22	N/A^*a*^
MTBC negative	23	23	N/A
NTM positive	N/A	N/A	17
NTM negative	N/A	N/A	28
Clinical samples (*n*_2_ = 35)			
MTBC positive	31	31	N/A
MTBC negative	4	4	N/A
NTM positive	N/A	N/A	0
NTM negative	N/A	N/A	35
Total	80		

^*a*^ N/A, not applicable.

In addition to MTBC detection, our method enabled NTM screening. Analysis of the proficiency testing panels identified 17 NTM-positive samples, while no NTM was detected in the clinical sample set (Table 4). To further evaluate the assay’s NTM screening capacity, we analyzed samples with suspected NTM infection, which were pre-characterized as mycobacterial strains via real-time PCR melting curve analysis (Table S1). The LFB–RT–LAMP assay demonstrated robust performance. When combining data from the accuracy testing strain panel (*n* = 35, Table 3) and these two groups of samples, the assay correctly identified 38 NTM positive and 77 NTM negative, achieving 100% agreement with the reference methods. The exact 95% confidence intervals were 90.8%–100% for sensitivity and 95.3%–100% for specificity.

## DISCUSSION

The persistent global burden of tuberculosis, coupled with the rising incidence of infections caused by NTM, presents a formidable diagnostic challenge for clinicians worldwide due to their overlapping clinical and radiological manifestations (1, 2, 7). This diagnostic dilemma often leads to delayed or incorrect treatment, exacerbating patient outcomes. Our study directly addresses the urgent need for novel diagnostic tools capable of accurate and rapid differentiation between these pathogens, as emphasized in the latest WHO consolidated guidelines on tuberculosis.

The WHO recommends moderate complexity automated nucleic acid amplification tests as a superior alternative to culture for the initial detection of pulmonary TB (24). However, a significant gap remains in the availability of such tests that can simultaneously detect and differentiate MTB from NTM in a single, rapid assay. While GeneXpert MTB/RIF and GeneXpert MTB/RIF Ultra are the cornerstones of modern TB diagnosis, they are primarily designed for MTB and rifampicin resistance detection and are unable to detect NTM species (25, 26). Our developed LFB–RT–LAMP platform aligns perfectly with the WHO’s strategic direction toward molecular testing and, crucially, advances the paradigm of “multi-disease testing” that is poised to replace outdated smear microscopy and single-disease assays (27).

The selection of LAMP as the foundational technology was strategic, capitalizing on its inherent advantages of operational simplicity, rapid reaction kinetics, and high specificity under isothermal conditions (12–14). These attributes make it exceptionally suitable for deployment in peripheral healthcare settings with limited infrastructure. In the present study, we leveraged the multi-primer characteristic of LAMP technology to implement multiple labeling strategies, enabling the same amplification products to be compatible with different detection methods and thus adaptable to diverse application scenarios. Specifically, for LFB detection, the amplicon only needs to contain the antigen region (capture zone) and a biotin-labeled segment for color development to be effectively detected (16, 22, 23). In the construction of the real-time fluorescence LAMP detection system, we introduced the labeling primarily on the LF/LB primers. This labeling design allows the strand-displacement process to interfere with the quenching effect of BHQ on the fluorophore, thereby releasing fluorescent signals for detection. This strategy differs significantly from those reported in current studies: our labeling system does not require specific base modifications for self-quenching (28), does not generate an inverted peak for signal readout (29, 30), and furthermore, eliminates the need for additional enzymes such as endonuclease or high-fidelity DNA polymerase to achieve cleavage (31–33).

In this study, labeling with antigens and fluorophore groups was primarily applied to the FIP/BIP and LF/LB primers, while the F3/B3 primers were generally not used for labeling. This is because LAMP amplicons are mainly generated from the extension of the inner primer pair (FIP/BIP), and the loop primers (LF/LB) further accelerate the overall LAMP amplification by displacing the stem-loop amplicons (12, 34). Thus, conventional labeling is typically introduced into the FIP/BIP and LF/LB primers, which are responsible for producing the major amplification products (16, 23, 28–33). To better demonstrate the performance of different labeling approaches, we labeled the LB primer of the *IS6110* gene with a FAM-BHQ1 fluorescence-quencher pair and the LF primer of the 16S rDNA gene with a Cy5-BHQ2 pair, illustrating the feasibility of this labeling strategy in practice (Table 2 and Fig. 2). Furthermore, by integrating the LFB detection strategy with the fluorescence labeling system, we further leveraged the multi-primer advantage of LAMP technology, enabling the same amplification products to be detected through different readout methods, thereby meeting the requirements of varied application scenarios.

The strengths of this study lie in the integration of LAMP technology, through which we constructed and validated a dual-modal detection platform based on multiple labeling that enables simultaneous LFB detection and real-time fluorescence detection, combined with dual-target gene analysis. This platform serves two main purposes: first, it expands the application scenarios of LAMP—the LFB is suitable for resource-limited settings or on-site rapid screening, while real-time fluorescence detection can be used for point-of-care testing and real-time monitoring, thereby offering a new paradigm for LAMP applications. Second, the dual-target combination is designed in light of the clinical profiles of TB and NTM-PD: since tuberculosis remains the predominant threat, the platform enables species-level identification of MTBC; for the highly diverse NTM, genus-level identification is performed. This combination is suitable for initial patient screening, as it can rapidly provide preliminary diagnostic guidance and inform medication choices, while also directing focus for subsequent more detailed diagnostics, such as tuberculosis drug susceptibility testing or species-level identification of NTM.

The Mycobacteria family comprises over 190 species, among which the MTBC is highly pathogenic, while a number of pathogenic NTM also exist (3, 4). Establishing a balanced and efficient diagnostic approach for these pathogens is of significant clinical importance. This study presents a comprehensive solution: by simultaneously targeting the MTBC-specific *IS6110* insertion sequence and the pan-mycobacterial 16S rDNA gene. Our method enables rapid discrimination of the MTBC while also confirming the presence of mycobacteria (including NTM), thereby providing key guidance for first-line treatment decisions within 60 min. Nevertheless, four main limitations remain in the current approach. First, the *IS6110* gene may be absent in some strains (35, 36). Second, a limitation of using pan-mycobacterial 16S rDNA for NTM screening is that it does not distinguish between different NTM species, and the target may not cover all mycobacterial species. Third, the method cannot yet reliably distinguish co-infection with both types of pathogens. Fourth, our assay has not been validated on extrapulmonary specimens (e.g., pleural fluid, cerebrospinal fluid), which are often paucibacillary; further studies are needed to assess its utility in such samples.

In this study, we used a commercial automated nucleic acid extractor and its reagents for DNA extraction from sputum samples. We acknowledge that reliance on such instrumentation remains a bottleneck for true POC deployment in resource-limited settings. However, LAMP is less susceptible to common PCR inhibitors than real-time PCR, making it compatible with crude DNA extracts. Mun et al. evaluated several lysis methods (sonication, 100°C for 20 min, 65°C for 30 min) and found all were suitable for downstream isothermal amplification (37). Notably, boiling extraction at 100°C for 10–20 min is a simple, reagent-free method that simultaneously inactivates viable mycobacteria, meeting standard biosafety requirements. Therefore, integrating a boiling-based extraction step with our LFB–RT–LAMP platform could enable POC testing without specialized extraction instruments or reagents, while preserving acceptable biosafety.

Another practical concern is the risk of amplicon contamination when transferring reaction products to the LFB. To mitigate this, our current workflow uses physically separated areas for reagent preparation, amplification, and detection, along with aerosol-resistant pipette tips and sealed reaction tubes. For LFB detection, we recommend decontaminating the work surface with chlorine-based disinfectant before and after each use. In resource-limited settings where LFB is used, testing can be performed in an open but well-ventilated area. Whenever contamination is a major concern, the real-time fluorescence module (closed tube) is the preferred mode because it does not require post-amplification handling. These measures and considerations are essential for translating our platform into field applications.

### Conclusion

In summary, our dual-modal LFB–RT–LAMP platform presents a novel LAMP strategy that integrates nano-biosensor-based readout with real-time fluorescence monitoring. This approach implements an innovative fluorescent probe labeling method for LAMP and demonstrates its feasibility. Based on this design, we have established a diagnostic workflow capable of simultaneously detecting MTBC (via the *IS6110* target) and screening for NTM (via the 16S rDNA target) in a single reaction. The method achieves a sensitivity of 576 fg per reaction (approximately 4.8 copies/μL) and maintains 100% specificity against non-mycobacterial pathogens. Clinical validation using 80 sputum samples, including challenging proficiency testing panels from the China CDC, further confirms its feasibility and accuracy in real-world settings. By offering flexible result output through either LFB strips or real-time fluorescence detection, the platform significantly expands its applicability across diverse scenarios. With moderate operational complexity and a turnaround time of less than 60 min, it demonstrates considerable potential for point-of-care use in both clinical and field environments.

## Data Availability

The raw sequence data used in this current study were obtained from GenBank under accession no. X98149.1 for *Mycobacterium tuberculosis IS6110*, right inverted repeat (https://www.ncbi.nlm.nih.gov/nuccore/X98149.1/), and accession no. MH794239.1 for *Mycobacterium tuberculosis* H37Rv 16S ribosomal RNA gene, partial sequence (https://www.ncbi.nlm.nih.gov/nuccore/MH794239.1/).

## SUPPLEMENTAL MATERIAL

Supplemental materials jcm.00662-26-s0001.pdf Supplemental materials and methods, Fig. S1 to S5, and Table S1.

ASM does not own the copyrights to Supplemental Material that may be linked to, or accessed through, an article. The authors have granted ASM a non-exclusive, world-wide license to publish the Supplemental Material files. Please contact the corresponding author directly for reuse.
