# Supplementary material for: Dual-modal LAMP platform integrating biosensor and real-time fluorescence for rapid differentiation of Mycobacterium tuberculosis complex from nontuberculous mycobacteria
Source: J Clin Microbiol. 2026 Sep 21;64(10):e00662-26. doi: 10.1128/jcm.00662-26 (PMC13712960; doi:10.1128/jcm.00662-26)
Supplement: Supplementary file 1 [file jcm.00662-26-s0001.pdf]

Supplementary Materials

Dual-modal LAMP Platform Integrating Biosensor and Real-time Fluorescence for Rapid Differentiation of *Mycobacterium Tuberculosis* Complex From *Non-tuberculous Mycobacteria*

Junfei Huang[1, 2†], Xiaoyu Wei[1, 2†], Yonghu Wan[1, 2], Chao yang[3], Fengming Chen[2], Lu Zou[2], Yijiang Chen[2], Jing Chen[4], Xiu He[1], Shijun Li[1, 2*], Mingdan You[1**], Guanghong Yang[1, 2***]

[1]School of Public Health, the Key Laboratory of Environmental Pollution Monitoring and Disease Control, Ministry of Education, Guizhou Medical University, Guiyang, Guizhou 561113, China;

[2]Guizhou Provincial Center for Disease Control and Prevention, Guiyang, Guizhou 550004, China;

[3]State Key Laboratory of Functions and Applications of Medicinal Plants, Guizhou Provincial Engineering Technology Research Center for Chemical Drug R&D, Guizhou Medical University, Guiyang, Guizhou 561113, China;

[4]Department of Nosocomial Infection Control, Guizhou Provincial People's Hospital, Guiyang, Guizhou 550002, China;

[†] These authors contributed equally to this work.

* **Correspondence:**

*Corresponding author.

**Corresponding author.

***Corresponding author. Guanghong Yang (School of Public Health, Guizhou Medical University, Guiyang, Guizhou 561113, China)

E-mail address: zjumedjun@163.com (Shijun Li), youmingdan@gmc.edu.cn (Mingdan You), ghyang_gzmu@outlook.com (Guanghong Yang).

Methods and materials

The GeneXpert MTB/RIF assay

Following the manufacturer's guidelines, sputum specimens were processed by mixing with the GeneXpert Sample Reagent at a 2:1 ratio (typically adjusted based on sputum viscosity). The mixture was vortexed thoroughly and incubated at room temperature for 15 minutes, with intermittent manual inversion to ensure complete sample liquefaction and mycobacterial inactivation. A 2 mL aliquot of the treated sample was subsequently transferred into the Xpert MTB/RIF assay cartridge, which integrates all necessary reagents for nucleic acid extraction and amplification. The cartridge was then loaded into the GeneXpert Instrument System for fully automated analysis. This system autonomously performs sample purification, concentration of *mycobacterium tuberculosis* complex (MTBC) bacteria, and subsequent real-time polymerase chain reaction (RT-PCR) amplification targeting the *rpoB* gene.

Molecular Identification of Mycobacteria Using Multicolor Melting Curve Analysis

The nucleic acid amplification reaction was performed using the MeltPro® Myco assay (Xiamen Zeesan Biotech, China), which is based on multicolor melting curve analysis (MMCA) technology. The reaction mixture with a final volume of 25 µL contained the extracted DNA template, specific primers, and fluorescently labeled probes targeting the internal transcribed spacer (ITS) region for species identification. To ensure procedural reliability, an exogenous internal control (Arabidopsis thaliana SUC2 gene) was incorporated into each reaction.

Amplification was carried out on a real-time PCR instrument (Shanghai Hongshi SLAN-48P, China) with the following protocol: initial denaturation at UNG treating 50°C for 2 minutes; 95°C for 10 minutes; 55 cycles of denaturation at 95°C for 5 seconds, annealing at 57°C for 20 seconds, and extension at 78°C for 20 seconds; incubation at 40°C for 30 minutes. Following amplification, melting curve analysis was immediately conducted by 95°C for 2 minutes, 40°C for 2 minutes, then gradually increasing the temperature from 40°C to 90°C at a rate of 0.04°C per second, with continuous fluorescence monitoring. Species identification was automatically determined by the instrument's software based on the characteristic melting temperature (Tm) of the probe-target hybrid, which is dictated by the specific sequence of each mycobacterial species.

This assay enables the simultaneous differentiation of 19 clinically relevant mycobacterial species or complexes, including but not limited to: *M. smegmatis*, *M. bovis*, *M. scrofulaceum*, *M. tuberculosis complex*, *M. chelonae*, *M. simiae*, *M. lentiflavum*, *M. gordonae*, *M. kansasii*, *M. abscessus*, *M. fortuitum*, *M. marinum (ulcerans)*, *M. terrae*, *M. nonchromogenicum*, *M. szulgai*, *M. malmoense*, *M. xenopi*, *M. intracellulare*, and *M. avium*.

Figures

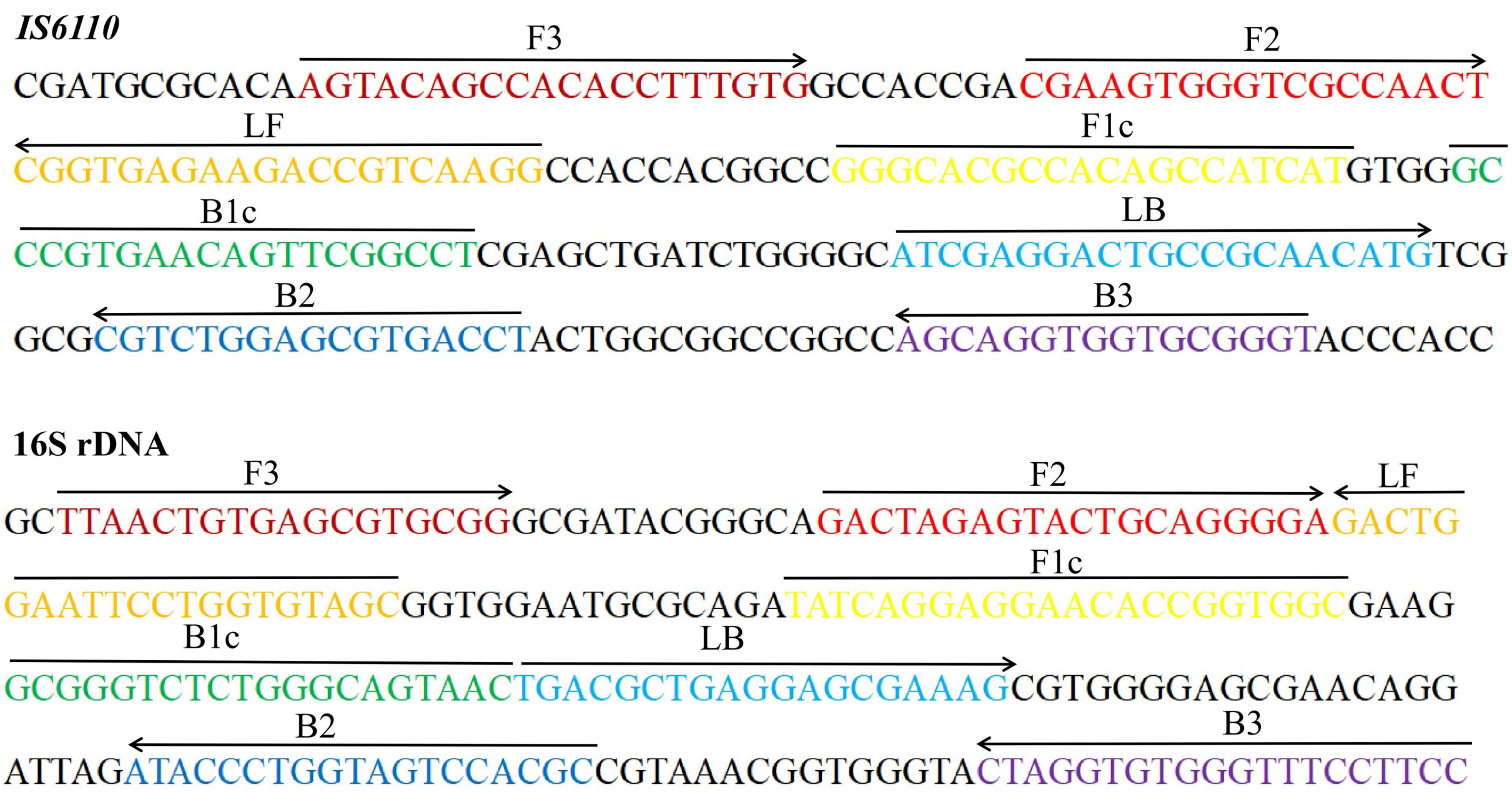

Figure S1 Primer design for dual LAMP assays targeting *IS6110* and 16S rDNA. The sense strand of each gene is shown, with primer-binding sites marked by arrows: rightward for sense primers, leftward for anti-sense primers. F3, forward outer primer; B3, backward outer primer (shown as its reverse complement). FIP, forward inner primer, comprises the reverse complement of F1c joined to F2. BIP, backward inner primer, comprises B1c joined to the backward complement of B2. LF and LB are loop primers. All primer sequences are listed in **Table1**.

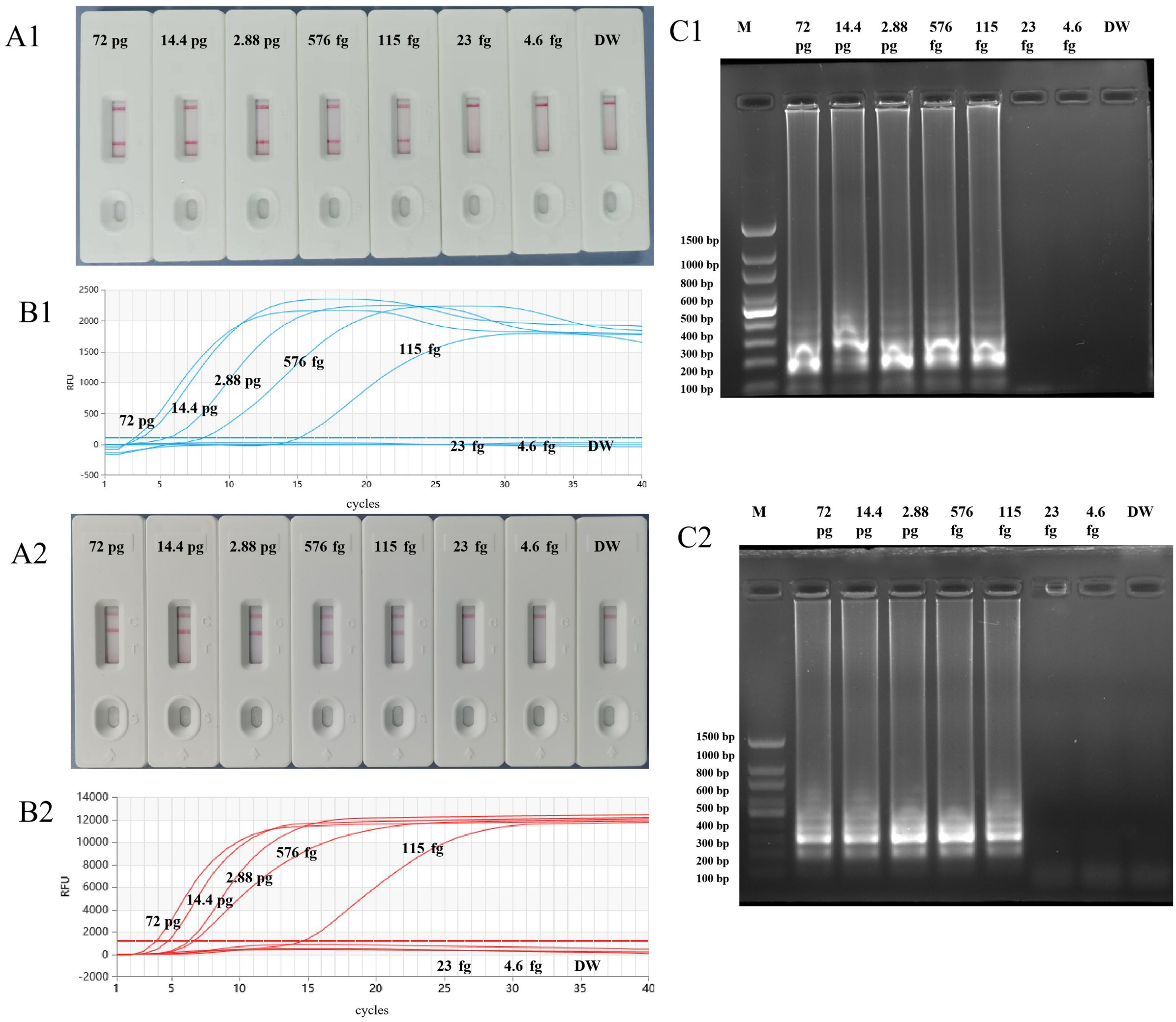

Figure S2 Detection threshold of the single-target LFB–RT–LAMP assay. Detection results for serial dilutions of H37Rv genomic DNA are shown for each target. **(A1, A2)** LFB readout for the *IS6110* (A1) and 16S rDNA (A2) targets. **(B1, B2)** Corresponding real-time fluorescence amplification curves for *IS6110* (B1) and 16S rDNA (B2). **(C1, C2)** Agarose gel electrophoresis analysis for *IS6110* (C1) and 16S rDNA (C2). **M**, DNA ladder marker.

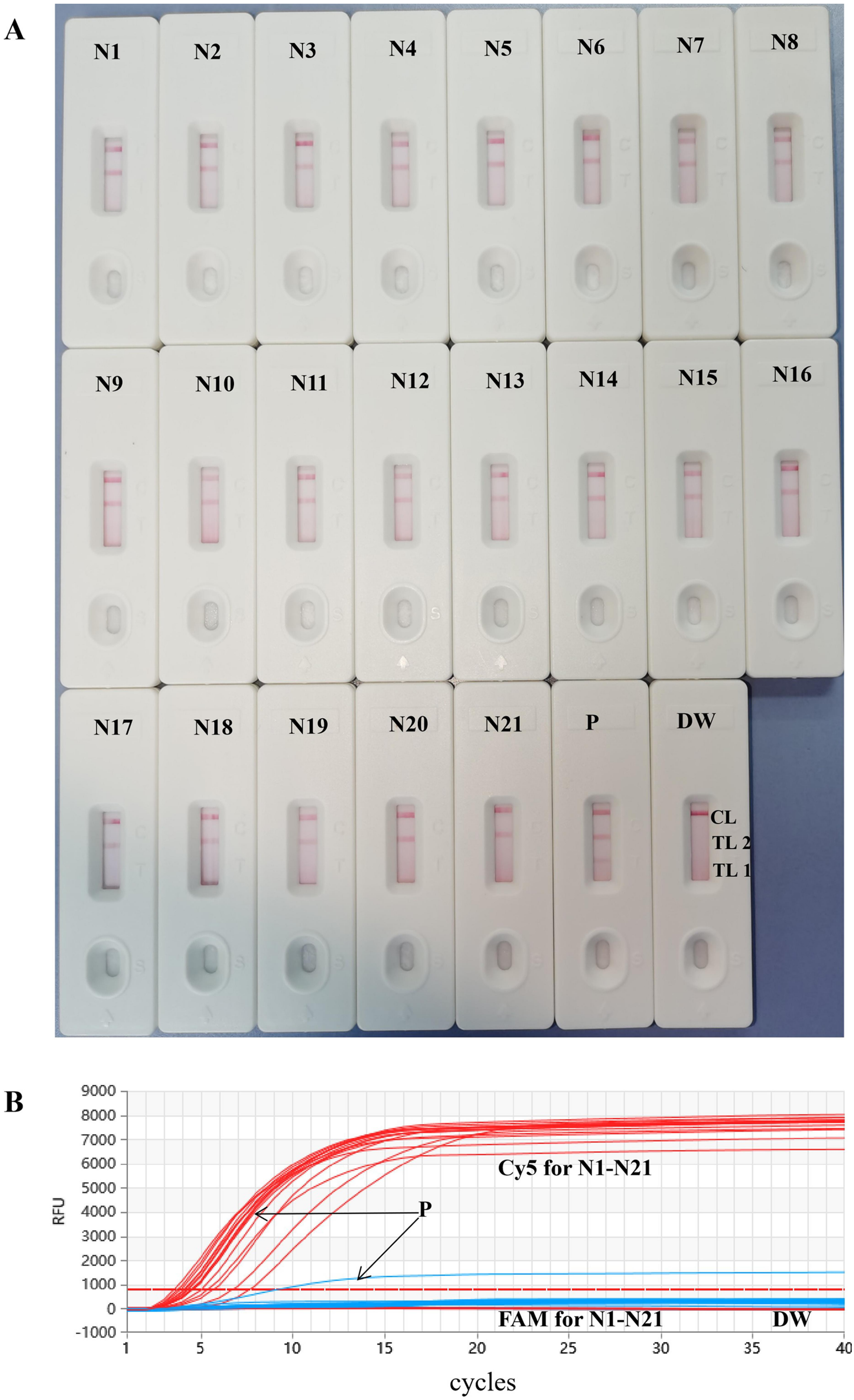
A
N1
N2
N3
N4
N5
N6
N7
N8
N9
N10
N11
N12
N13
N14
N15
N16
N17
N18
N19
N20
N21
P
DW
CL
TL 2
TL 1
B
RFU
9000
8000
7000
6000
5000
4000
3000
2000
1000
0
-1000
1
5
10
15
20
25
30
35
40
cycles
Cy5 for N1-N21
P
FAM for N1-N21
DW

77

Figure S3 Capacity of the LFB–RT–LAMP assay to identify clinically relevant mycobacterial species. The assay was performed using a panel of mycobacterial genomic DNA templates and monitored by two methods. **(A)** LFB readout. **(B)** Real-time fluorescence monitoring. The following strains (N1–N21) were tested: *M. kansasii, M. intracellulare, M. chelonae, M. neoaurum, M. marinum, M. gilvum, M. microti, M. vaccae, M. phlei, M. scrofulaceum, M. gastri, M. xenopi, M. abscessus, M. ulcerans, M. malmoense, M. leprae, M. avium, M. intracellulare, M. fortuitum, M. chelonae, and M. smegmatis*. **P**: Positive control (*M. tuberculosis* H37Rv). **DW**: No-template control (DNase-free water).

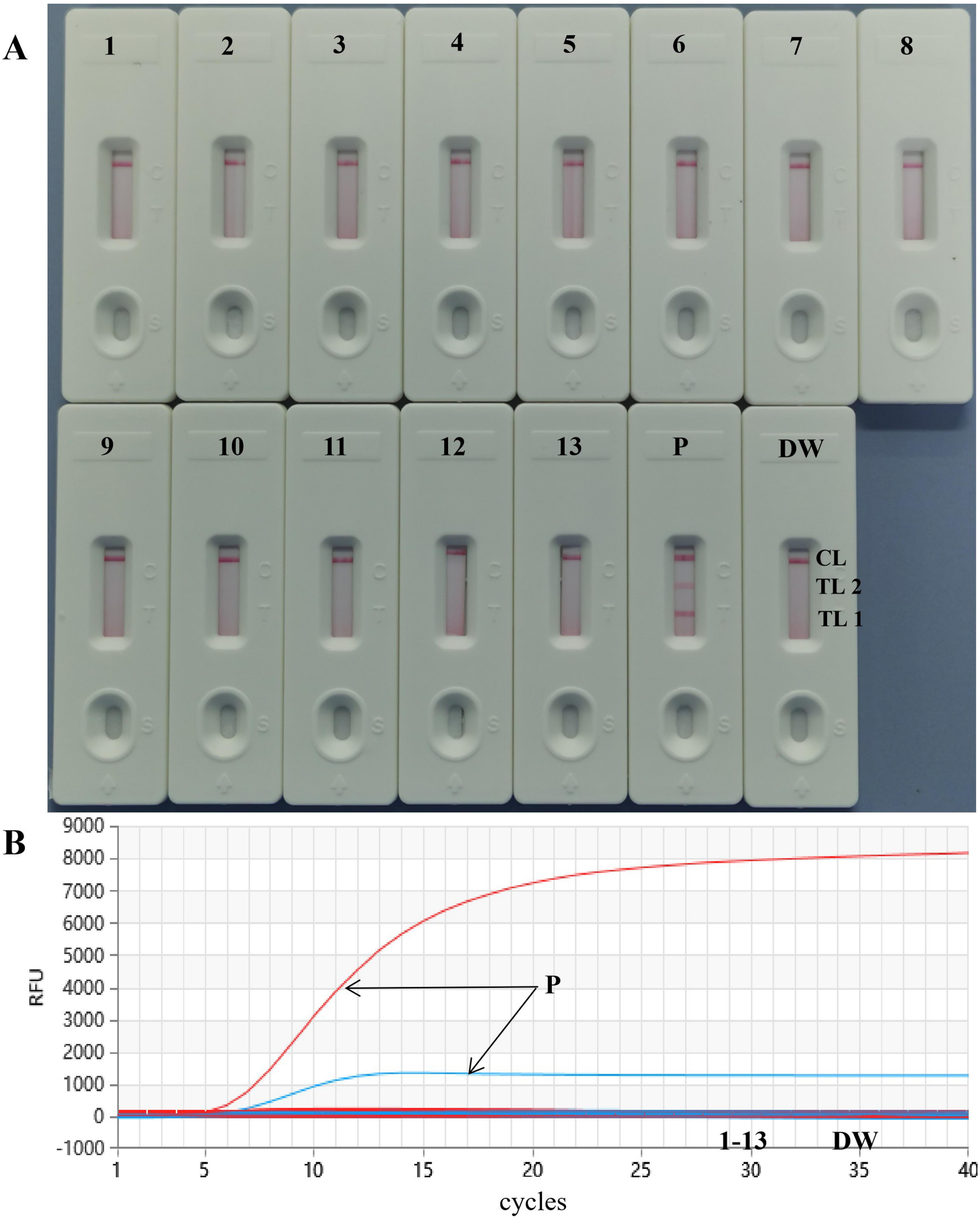

Figure S4 Assessment of LFB–RT–LAMP assay cross-reactivity with non-mycobacterial pathogens. The assay was tested against the clinically relevant non-mycobacterial pathogens. **(A)** LFB readout. **(B)** Real-time fluorescence monitoring. **1–13**, correspond to the following pathogens: *Brucella melitensis, Klebsiella pneumoniae, Haemophilus influenzae, Streptococcus pneumoniae, Staphylococcus aureus, nontyphoidal Salmonella, Pseudomonas aeruginosa, Bacillus anthracis, Streptococcus suis, Neisseria gonorrhoeae, Shigella dysenteriae,* HIV-1, and

H1N1. **P**: Positive control (*M. tuberculosis* H37Rv). **DW**: No-template control (DNase-free water).

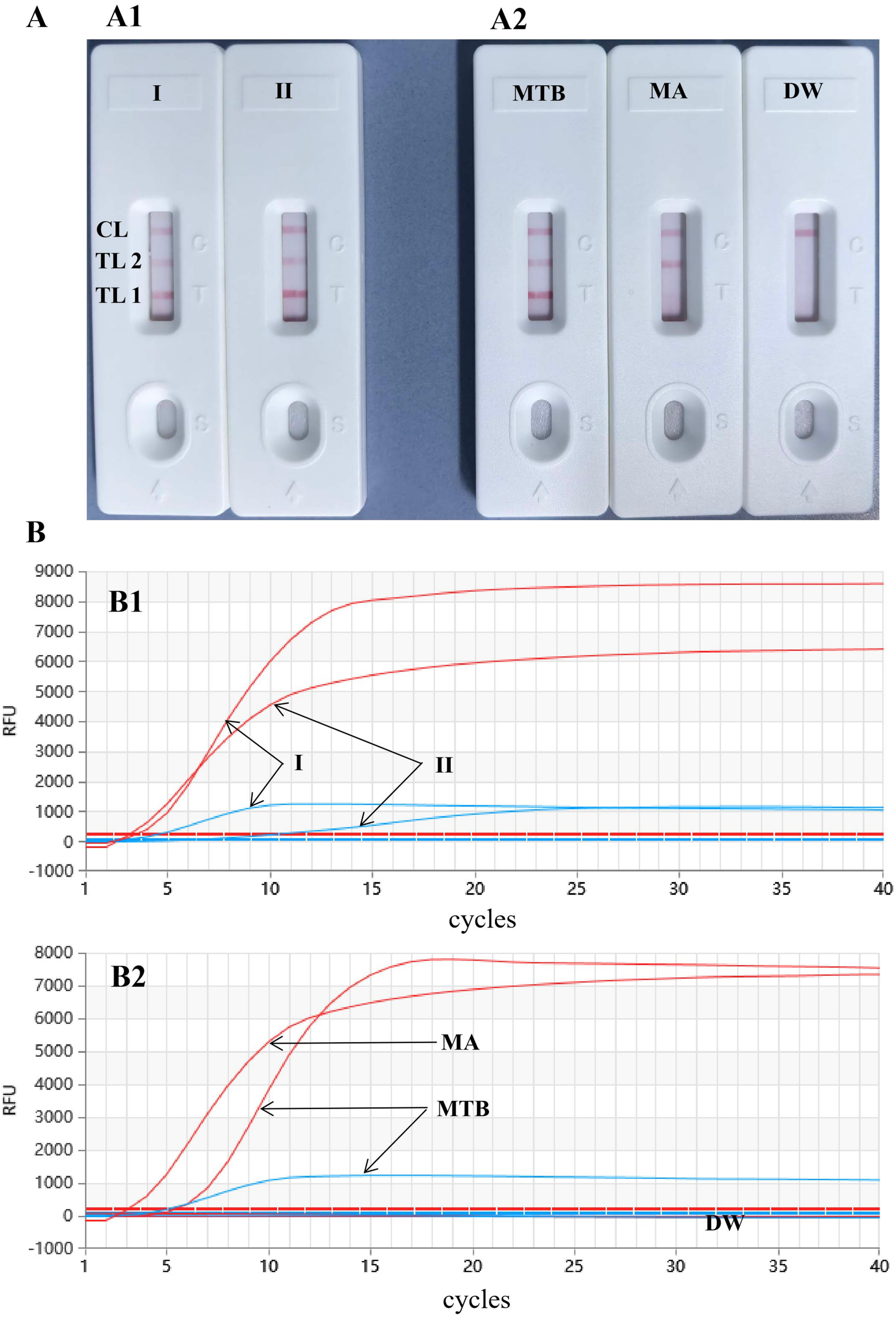

Figure S5 Assessment of MTBC-NTM co-infection detection using the LFB–RT–LAMP assay. **(A)** LFB readout. **(B)** Real-time fluorescence amplification curves (blue: FAM-IS6110; red: Cy5-16S rDNA). **I**: MTB (72 pg) mixed with *M. avium* (MA) DNA; both *IS6110* (TL1/FAM) and

16S rDNA (TL2/Cy5) targets are detected. **II:** MTB (576 fg, LoD) mixed with MA DNA; both targets are also detected. **Controls**: **MTB** only (14.4 pg, both targets detected), **MA** only (16S rDNA positive, IS6110 negative), and DNase-free water (both targets negative). In all co-infection samples, MTB detection remained unaffected by the presence of MA.

Tables

Table S1 Detection results for NTM-suspicion samples by real-time PCR melting curve analysis and LFB-RT-LAMP assay

**Method**	**Real-time PCR Melting Curve Analysis [a]**	**LFB-RT-LAMP [b]**			
**NO.**		**TL1**	**TL2**	**FAM**	**CY5**
2022-M10	*Mycobacterium*	-	+	-	+
2022-N2	*M. avium*	-	+	-	+
2022-N3	*M. kansassi*	-	+	-	+
2022-N4	*M. abscessus*	-	+	-	+
2022-N5	*Mycobacterium*	-	+	-	+
2023-M9	*M. kansassi*	-	+	-	+
2023-N1	*M. intracellulare*	-	+	-	+
2023-N2	*Mycobacterium*	-	+	-	+
2023-N3	*M. smegmatis*	-	+	-	+
2023-N4	*Mycobacterium*	-	+	-	+
2023-N5	*M. abscessus*	-	+	-	+
2024-M9	*M. abscessus*	-	+	-	+
2024-N1	*M. intracellulare*	-	+	-	+
2024-N2	*M. fortuitum*	-	+	-	+
2024-N3	*M. gordonae*	-	+	-	+
2024-N4	*M. avium*	-	+	-	+
2024-N5	*M. abscessus*	-	+	-	+

Notes: a The reference real-time PCR melting curve analysis can identify 19 mycobacterial species. Samples with melting profiles outside the defined ranges for these species are reported only to the genus level (Mycobacterium spp.). **b** For the LFB-RT-LAMP assay: TL1/FAM targets the *IS6110* gene; TL2/Cy5 targets the 16S rDNA gene. +, positive; -, negative.
